# Draft Genome Sequence of Beneficial Rice Rhizosphere Isolate *Pseudomonas aeruginosa* PUPa3

**DOI:** 10.1128/genomeA.00654-14

**Published:** 2014-07-03

**Authors:** Gordana Uzelac, Iris Bertani, Milan Kojic, Konrad H. Paszkiewicz, David J. Studholme, Daniel Passos da Silva, Vittorio Venturi

**Affiliations:** a International Centre for Genetic Engineering & Biotechnology, Trieste, Italy; b Institute of Molecular Genetics and Genetic Engineering, University of Belgrade, Belgrade, Serbia; c Biosciences, College of Life and Environmental Sciences, University of Exeter, Exeter, Devon, United Kingdom

## Abstract

*Pseudomonas aeruginosa* PUPa3 is a rhizosphere-colonizing and plant growth-promoting strain isolated from the rhizosphere of rice. This strain has, however, been shown to be pathogenic in two nonmammalian infection models. Here we report the draft genome sequence of *P. aeruginosa* PUPa3.

## GENOME ANNOUNCEMENT

*Pseudomonas aeruginosa* is a metabolically versatile Gram-negative bacterium that is mostly studied as an opportunistic human pathogen causing chronic infections in the lungs of cystic fibrosis patients (1, 2). *P. aeruginosa* is thought to be ubiquitous in nature and can be found in aquatic sediments, water-exposed surfaces, soil, plant roots and leaves, and also on human and animal surfaces (3, 4). This bacterium has numerous signaling regulatory pathways responsible for sensing, responding, and adapting to many different environmental conditions (5). It remains unclear whether there are significant genomic differences between environmental and clinical *P. aeruginosa* isolates. Plant environments are believed to be reservoirs for several human opportunistic pathogens including *P. aeruginosa* (6).

*P. aeruginosa* strain PUPa3 is a plant growth-promoting strain with a broad spectrum of antifungal activity and was isolated from the rhizosphere of rice in India (7). PUPa3, like *P. aeruginosa* PAO1, was reported to have two different *N*-acyl homoserine lactone (AHL)-dependent quorum-sensing (QS) systems, namely, the LasI/R and RhlI/R systems. Strain PUPa3 has been shown to be virulent in the *Caenorhabditis elegans* and the wax moth *Galleria mellonella* infection models, and the two AHL systems are required for full virulence. Interestingly, unlike what occurs in strain PAO1, the two QS systems are not hierarchically arranged and do not cross-regulate each other and thus act independently (8).

Here we announce the draft genome sequence of beneficial rhizosphere-colonizing *P. aeruginosa* PUPa3. The genome sequence of *P. aeruginosa* PUPa3 was determined using a 150-bp paired-end library with the Illumina Hi Seq 2500 system. The total number of pairs of reads was 5,748,337, representing approximately 135-fold coverage of the genome. We performed the *de novo* assembly using Velvet 1.2.09 (9), generating 138 contigs with a maximum length of 723 kbp. The total length of the contig assembly was 6.3 Mbp, and the *N*_50_ length was 208 kbp, assuming a genome size of 6.3 Mbp. The G+C content was 65.8%, similar to that of other *Pseudomonas* sequenced genomes. Automated annotation of the *P. aeruginosa* PUPa3 draft genome sequence using Prokka 1.7 (10) assigned a total of 5,838 candidate protein-coding genes. A total of 4 rRNA and 59 tRNA genes were identified by the Prokka annotation system. Comparative genome analysis was performed on the *P. aeruginosa* PAO1 genome (GenBank accession no. NC_002516.2) using MUMmer (11) and it was found that 96% of PAO1 is aligned with PUPa3 with an average of 99% identity.

### Nucleotide sequence accession numbers.

This whole-genome shotgun project has been deposited at DDBJ/EMBL/GenBank under the accession no. JJOD00000000. The version described in this paper is the first version, number JJOD01000000.
